# First Steps in the Development of a New Measure of Attitudes Toward Sexual Offending Against Children

**DOI:** 10.5964/sotrap.11895

**Published:** 2023-12-20

**Authors:** Kevin L. Nunes, Danielle M. L. Hawthorn, Emily R. Bateman, Amy L. Griffith, Julia M. Fraser

**Affiliations:** 1 Department of Psychology, Carleton University, Ottawa, Canada; 2 Missouri Sexual Offense Services, Centurion, Jefferson City, MO, USA; University of Lincoln, Lincoln, United Kingdom

**Keywords:** evaluative attitudes, sexual offending against children, measurement

## Abstract

It is unclear whether existing measures of attitudes and cognitive distortions regarding sexual offending against children (SOC) reflect evaluative attitudes toward SOC (i.e., how negatively or positively one views SOC). The purpose of the current study was to take the first steps toward creating a self-report measure of evaluative attitudes toward SOC. We created 30 items and asked 157 incarcerated people in a sexual offense treatment program to complete them. We retained the 13 items that were the least positively skewed (i.e., lowest endorsement of the most negative response option) and non-redundant (i.e., not too highly correlated with other items) for inclusion in the new measure, which we called the Evaluative Attitudes Toward Sexual Offending Against Children (EASOC) Scale. As an initial test of the relevance of the EASOC Scale, we examined its association with SOC. Participants with SOC (n = 58) reported more positive evaluative attitudes on the EASOC Scale than did those without SOC (n = 22). This expected association is a necessary (but not sufficient) indication that the EASOC Scale may be relevant for predicting and explaining SOC. Future research using more rigorous methodology should build on our modest first steps to revisit item selection and test the validity and relevance of the EASOC Scale.

## Highlights

- Evaluative attitudes toward sexual offending against children (SOC) refers to how negatively or positively one views SOC.
- We created a 13-item self-report scale designed to measure evaluative attitudes toward SOC: Evaluative Attitudes Toward Sexual Offending Against Children (EASOC) Scale.
- Participants who had committed SOC reported more positive evaluative attitudes on the EASOC Scale than did those who had not committed SOC.
- Our preliminary findings support further research on the EASOC Scale with more rigorous methodologies.

The terms *attitudes* and *cognitive distortions* have often been used interchangeably in the sexual aggression literature (e.g., Helmus et al., 2013; Nunes et al., 2018; Ó Ciardha & Ward, 2013). Though definitions have been fuzzy and inconsistent (e.g., Nunes et al., 2018; Ó Ciardha & Ward, 2013; Szumski et al., 2018), both attitudes and cognitive distortions often appear to be used—explicitly or implicitly—to refer to a wide range of thoughts and beliefs that may condone, justify, excuse, minimize, rationalize, or otherwise support sexual offending (e.g., Abel et al., 1989; Bumby, 1996; Gannon & Polaschek, 2006; Gannon et al., 2007; Hanson et al., 1994; Mann & Beech, 2003; Mann, Hanson, & Thornton, 2010; Maruna & Mann, 2006; Nunes et al., 2018; Ó Ciardha & Gannon, 2011; Ó Ciardha & Ward, 2013; Szumski et al., 2018; Ward et al., 2006). Attitudes and cognitive distortions have been hypothesized in theories to play a causal role in sexual offending (for reviews, see Ó Ciardha & Ward, 2013, and Szumski et al., 2018), generally have been found to correlate with and predict sexual offending (e.g., Helmus et al., 2013; Hermann & Nunes, 2018; Nunes et al., 2013, 2018; Pedneault et al., 2021, 2022; Whitaker et al., 2008), and are often addressed in risk assessment (e.g., Olver et al., 2007) and treatment (e.g., McGrath et al., 2010) aimed at reducing further sexual offending.

Recent work has compared and contrasted definitions and measurement of attitudes in the forensic/correctional/criminological domain versus the general social psychological literature (Nunes et al., 2015, 2018, 2021, 2023; Pedneault et al., 2021). As noted above, in the forensic domain, attitudes and cognitive distortions have often been used interchangeably to refer to a wide range of cognitions. In the general social psychological literature, however, attitudes are typically defined as evaluations (e.g., bad vs. good) of psychological objects, such as behaviors. In their research on violence, Nunes et al. (2021) used the term *evaluative attitudes* to refer to the extent to which violence is viewed negatively or positively (consistent with the definition of attitudes in the social psychological literature), to distinguish it from the broader lay definition of *attitudes* that often seems to be used in the forensic area. Recent research has developed measures of evaluative attitudes toward sexual aggression against women and found evidence that suggests they may be distinct from cognitive distortions and that both evaluative attitudes and cognitive distortions are independently associated with sexual aggression (Hermann & Nunes, 2018; Nunes et al., 2018; Pedneault et al., 2021).

It is similarly unclear whether existing measures of attitudes and cognitive distortions regarding sexual offending against children (SOC) reflect evaluative attitudes toward SOC. Consider the items selected from various cognitive distortions measures in Table 1.

**Table 1:** Sample Items From Self-Report Scales of Cognitive Distortions Regarding Sexual Offending Against Children

Item
A child 13 or younger can make her (his) own decisions as to whether she (he) wants to have sex with an adult or not^a^
Most children 13 (or younger) would enjoy having sex with an adult, and it wouldn’t harm the child in the future^a^
Sometime in the future, our society will realize that sex between a child and an adult is all right^a^
An adult just feeling a child’s body all over without touching her (his) genitals is not really being sexual with the child^a^
I show my love and affection to a child by having sex with her (him)^a^
Some children are so willing to have sex that it is difficult to stay away from them^b^
The innocent look of young girls makes them attractive^b^
As long as the child does not protest, it is OK to touch his or her genitals^b^
Some sexual relations with children are a lot like adult sexual relationships^c^
Kids who get molested by more than one person are probably doing something to attract adults to them^c^
Some children are willing and eager to have sexual activity with adults^c^
I think the main thing wrong with sexual activity with children is that it is against the law^c^
When adults and children have sexual relationships it’s not always the adults’ fault^d^
If children want, they should be allowed to have sexual relationships with adults^d^
Having sex with a child is not really all that bad because it doesn’t really harm the child^e^
Having sex with a child is a good way to teach them about sexuality^e^

*Note.* For all of these measures, respondents rate the extent to which they agree or disagree with each item.

^a^Abel-Becker Cognition Scale (ABCS; Abel et al., 1989). ^b^Hanson Sex Attitudes Questionnaire (HSAQ; Hanson et al., 1994). ^c^MOLEST Scale (Bumby, 1996). ^d^Children and Sex Cognitive Distortions (CSCD) scale (Beckett, Beech, & Fisher, 1996). ^e^Sex With Children (SWCH) scale (Mann et al., 2007).

On one hand, some of the items may well directly reflect evaluative attitudes or could form—at least partially—the basis of one’s evaluative attitudes toward SOC. For example, viewing children as sexually attractive and believing that children want, enjoy, are not harmed by, and can give informed consent to sexual contact with adults would likely be associated with a positive evaluative attitude toward SOC. On the other hand, many of these features seem neither necessary nor sufficient indicators of evaluative attitudes toward SOC. That is, it seems possible that someone could view SOC as positive, but nevertheless not endorse many of these items because their evaluative attitudes are informed by different considerations or in different ways. For example, one may be indifferent to the victim’s experience or even enjoy the victim’s suffering, in which case beliefs that children can consent, enjoy, and not be harmed by sexual contact with adults could be unrelated or even negatively related to their evaluative attitudes. Further, it seems impossible to catalogue all the specific considerations that could form the bases of one’s evaluative attitudes toward SOC. Though there are likely some basic considerations common to most people, there are undoubtedly many more idiosyncratic considerations. Thus, evaluative attitudes toward SOC could be dissociated from the constructs measured by at least some items of typical cognitive distortion scales.

Extrapolating from the general social psychological literature (e.g., Ajzen, 1991, 2001; Fazio, 1990; Glasman & Albarracín, 2006; Kraus, 1995) and research on general violence and sexual aggression against women (e.g., Anderson & Bushman, 2002; Hermann & Nunes, 2018; Nunes et al., 2013, 2015, 2018, 2021, 2022, 2023; Pedneault et al., 2021, 2022), we expect that evaluative attitudes toward sexual offending against children would provide important complementary information for explaining, predicting, and reducing sexual offending against children. The purpose of the current study was to take the first steps toward developing a measure of evaluative attitudes toward sexual offending against children. We created several items and asked incarcerated people in a sexual offense treatment program to complete them. Based on their responses, we retained the least positively skewed (i.e., items for which the smallest proportion of participants gave the most negative response) and non-redundant items (i.e., items that were not too highly correlated with each other) for inclusion in the new measure, which we called the Evaluative Attitudes Toward Sexual Offending Against Children (EASOC) Scale. We then compared EASOC Scale scores between participants who reported a record of sexual offenses against children and participants who reported no record of sexual offenses against children. This comparison was meant to be an initial test of the EASOC Scale’s potential relevance for predicting and explaining sexual offending against children. Specifically, finding the expected difference between groups would be consistent with the possibility of relevance and support continued research on the EASOC Scale, whereas failing to find the expected difference between groups would suggest the EASOC Scale may not be relevant or worthy of continued research.

## Method

### Participants

People incarcerated in a Missouri state men’s prison and currently participating in a sex offender treatment program were recruited to participate in our study. The study was approved by the Carleton University Research Ethics Board – B (Clearance #112589) and permission was granted by the Missouri Department of Corrections. Of the 201 participants who started the questionnaire, we excluded data from participants who did not correctly answer the attention check items (*n* = 37, 18.4%) or whose response pattern suggested invalid data (*n* = 4, 2.0%). Of the remaining participants, data were also excluded from three participants (1.9%) who did not answer all the potential EASOC Scale items. This created a final sample size of 157 participants.

Participants’ mean age was 40.2 years (*SD* = 11.4) and the majority identified as men (144, 91.7%; 2, 1.3% other; 11, 7.0% missing). The racial composition of the sample was 122 (77.7%) White, 25 (15.9%) Black, 5 (3.2%) Indigenous, 4 (2.5%) Hispanic, and 2 (1.2%) other. In terms of current offenses, 3 (1.9%) had a violent offense, 4 (2.5%) had a nonviolent offense, 11 (7.0%) had a sexual offense against a person 18 years or older, 14 (8.9%) had a sexual offense against a 16 to 17 year old, 60 (38.2%) had a sexual offense against a 13 to 15 year old, 58 (36.9%) had a sexual offense against a person 12 years or younger, 20 (12.7%) had a child pornography offense, and 3 (1.9%) provided no information about current offenses. For prior offenses, 14 (8.9%) had a violent offense, 33 (21.0%) had a nonviolent offense, 1 (0.6%) had a sexual offense against a person 18 years or older, 2 (1.3%) had a sexual offense against a 16 to 17 year old, 5 (3.2%) had a sexual offense against a 13 to 15 year old, 9 (5.7%) had a sexual offense against a person 12 years or younger, 3 (1.9%) had a child pornography offense, 94 (59.9%) had no prior offenses, and 6 (3.8%) provided no information about prior offenses.

### Materials

Participants were presented with a paper-and-pencil questionnaire package with questions about their demographic characteristics, offense history, and evaluative attitudes toward sexual offending against children (the full questionnaire package is available in the Supplementary Materials). The demographic questions asked about age, gender, and race. The offense history questions asked about the nature of their current offenses (What kind of offenses are you currently in prison for?) and prior offenses (Not counting the offenses that you’re in prison for now, what kind of offenses were you convicted of before?). For each offense question, participants were instructed to select all of the following that applied: Violent (for example, assault, robbery, making threats), Non-violent non-sexual (for example, theft, break and enter, drug possession), Sexual against a person age 18 years or older (for example, sexual assault), Sexual against a 16 to 17 year old person, Sexual against a 13 to 15 year old person, Sexual against a person age 12 years or younger, Child pornography, or No prior offenses.

The remainder of the questionnaire contained 60 items designed to measure evaluative attitudes toward sexual offending against children and two attention check items designed to indicate whether participants were understanding and attending to the questionnaire. The 60 evaluative attitude items consisted of 30 statements presented with each of two response scales: once with *very bad*, *pretty bad*, *not so bad*, *not bad at all* and again with *very negative*, *pretty negative*, *not so negative*, *not negative at all*. Our use of *bad* and *negative* response scales followed the approach of research on evaluative attitudes toward sexual aggression against women and general violence (Nunes et al., 2015, 2018, 2021; Pedneault, 2021). One attention check question was embedded within each set of 30 evaluative attitude items (Please circle “not so bad”; Please circle “not so negative”). To minimize the potential influence of any one particular order of presentation, we created four different questionnaire orders, which consisted of two fixed random orders of the 30 evaluative attitude items and one attention check item, with each random order presented with either the *bad* response scale first or with the *negative* response scale presented first.

We created the items guided by the literature on attitudes in general (e.g., Eagly & Chaiken, 2007; Fazio, 2007; Gawronski & Bodenhausen, 2007), evaluative attitudes toward violence (Nunes et al., 2015, 2021), evaluative attitudes toward sexual aggression against women (Nunes et al., 2018; Pedneault, 2021), and cognitions regarding sexual offending against children (e.g., Abel et al., 1989; Gannon & Polaschek, 2006; Gannon et al., 2007; Ó Ciardha & Ward, 2013; Szumski et al., 2018; Ward et al., 2006). We went through several rounds of review, discussion, and revision among ourselves and the students in the first author’s lab to create an initial pool of items. We received feedback on our initial items from four colleagues with clinical and research expertise in the area of cognitions regarding sexual offending against children and we made further revisions to create the final set of items that we presented to participants. Our goal was to create evaluative attitude items sensitive enough to capture variance, but that were also unambiguously about adult-child sexual contact. That is, we aimed to minimize floor effects in which everyone or almost everyone would give the most negative response (“very bad/very negative”), but we avoided doing this at the expense of presenting behavior that did not indisputably reflect adult-child sexual contact that would constitute a sexual crime in most jurisdictions. Thus, we asked specifically about sexual contact between a child (13 years or younger) and adult (e.g., you sexually touch a child…), and, when not already clear, we explicitly specified the sexual nature/motivation of the adult’s behavior (e.g., …for your sexual pleasure).

We expected that people would be very unlikely to report anything other than the most negative evaluation of sexual offending against children. Thus, in an effort to maximize variance, we mentioned that answering the questions was not an indication that they had committed or would commit sexual offenses against children. Further, we focused on relatively less severe/intrusive sexual acts (e.g., sexual touching rather than penetration), used abstract wording to describe the sexual activity, made no explicit mention of coercive tactics, did not mention anything about negative reactions from or effects on the child, and, for most items, included contexts or features that we expected might be perceived as mitigating by some participants (e.g., the child initiates the interaction, opportunity presents itself, benevolent motivation). These potentially mitigating contexts were drawn largely from the cognitive distortions literature. However, in contrast to typical cognitive distortion scale items, we focused on evaluation of adult-child sexual contact in those contexts rather than on agreement with the extent to which those contexts or features are true or characteristic of adult-child sexual contact. For example, rather than asking participants whether they believe that children initiate sexual contact with adults (as in the typical cognitive distortions scale), we asked how bad it would be to sexually offend against a child who initiated the contact. Furthermore, rather than the typical symmetrical response scale (e.g., very bad vs. very good), we skewed the response scale to make it shades of negative: *very bad*, *pretty bad*, *not so bad*, *not bad at all*. Of course, our efforts to maximize variance were not intended to convince anyone that adult-child sexual contact is ever not a bad thing. Rather, the items must be sensitive enough for the measure to detect inter- and intra-individual differences and be suitable for statistical analyses. We also included one item with no context to serve as a baseline (*You sexually touch a child*).

### Procedure

Participants in the sex offense treatment program were told about the study and invited to participate. Recruitment and data collection was conducted by the fourth author, who, in her role as Clinical Director, oversees all assessment and treatment services for individuals incarcerated for sexual offenses in multiple institutions throughout the state. They were given the consent form, questionnaire package, debriefing form, and an envelope. The four different order versions of the questionnaire were randomly distributed. The people were instructed to complete the questionnaire package (or leave it blank if they did not wish to participate), place it in the envelope, seal the envelope, and hand it to the researcher. Data were anonymous. After data collection, we decided that the *bad* response scale was preferable to the *negative* response scale, because it is simpler, clearer, and more intuitive for participants. In hindsight, it seems more straightforward to ask someone if they think sexual offending is *bad* rather than if they think it is *negative*. This decision was based on feedback from colleagues, awareness of the low average reading level among people in prison, and concern that less clarity would result in more error. Thus, we considered only the *bad* response scale items for inclusion in the EASOC Scale (but see Table S1 in the Supplementary Materials for the frequencies of the *negative* response scale items).

## Results

### Item Reduction

Following the approach in past research on sexual aggression against women and general violence (e.g., Nunes et al., 2021; Pedneault, 2021), we aimed to reduce the pool of 30 items with the goal of minimizing floor effects and redundancy. Our first step was to identify any severely skewed items to which participants rarely responded with anything other than *very bad*. We started by ranking the items from lowest to highest percentage of *very bad* responses (see Table 2). We retained the 14 items with the lowest percentage of *very bad* responses (90.4% to 93.0%) and dropped the remaining 16 items with higher percentages (93.6% to 97.5%).

**Table 2:** Response Frequencies for Initial EASOC Scale Items

Item	Response			
	Very bad	Pretty bad	Not so bad	Not bad at all
A child puts their hand on your penis, and you let it continue for your sexual pleasure.	90.4%	7.0%	1.9%	0.6%
You let a child touch your penis for their sexual pleasure.	91.1%	6.4%	1.9%	0.6%
You are helping a child into their bathing suit, and you touch their genitals for your sexual pleasure.	91.1%	6.4%	2.5%	0.0%
A child sits on your lap and rubs against your penis, you let it continue for your sexual pleasure.	91.1%	5.7%	2.5%	0.6%
You are bathing a child and they ask you to wash their private parts. You wash and continue to rub their genitals to make the child feel good.	91.7%	4.5%	2.5%	1.3%
A child wants to touch your penis, and you let them for your sexual pleasure.	91.7%	5.1%	2.5%	0.6%
Your partner wants to sexually touch a child with you, so you do it to make your partner happy.	91.7%	6.4%	1.9%	0.0%
A child asks you about masturbation so you touch their genitals to teach them how to masturbate.	92.4%	5.1%	1.9%	0.6%
You sexually touch a child because they won’t reject you like adults will.	92.4%	6.4%	1.3%	0.0%
You have a ‘tickle fight’ with a child and touch their genitals for your sexual pleasure.	92.4%	4.5%	2.5%	0.6%
You sexually touch a child.	93.0%	5.1%	1.9%	0.0%
A child asks you about masturbation so you masturbate in front of the child to teach them how to do it.	93.0%	3.8%	2.5%	0.6%
You sexually touch a child to help them feel better about their body.	93.0%	6.4%	0.0%	0.6%
While you’re drunk, you sexually touch a child.	93.0%	5.7%	0.6%	0.6%
You have an erection as you hug a child. You continue to hug the child, pressing your penis against the child.	93.6%	4.5%	1.3%	0.6%
You are lonely, so you sexually touch a child to feel close to someone.	93.6%	4.5%	1.3%	0.6%
A child asks you about sex, so you sexually touch the child to teach them.	94.3%	3.8%	1.9%	0.0%
A child asks you what a penis is, so you show them yours and have them touch it for your sexual pleasure.	94.3%	3.8%	0.6%	1.3%
To prepare them for the adult world, you get two children to sexually touch each other while you watch.	94.3%	3.2%	1.9%	0.6%
You sexually touch a child who doesn’t have a lot of friends so that they can enjoy some attention.	94.9%	3.2%	1.3%	0.6%
You see that a child is sad, so you sexually touch them to cheer them up.	94.9%	3.8%	1.3%	0.0%
You sexually touch a child who is passed out.	94.9%	3.8%	0.6%	0.6%
You are very depressed, so you sexually touch a child to try to feel better.	94.9%	3.2%	1.9%	0.0%
You sexually touch a child who is sleeping deeply.	95.5%	2.5%	1.3%	0.6%
You sexually touch a child who you know has been neglected or abused, to show them love.	96.2%	3.2%	0.0%	0.6%
You tuck a child into bed at night and touch them sexually to help them get to sleep.	96.2%	3.2%	0.6%	0.0%
You are so horny that you sexually touch a child who is close by.	96.2%	2.5%	1.3%	0.0%
You sexually touch a child because their parents are neglecting them, and you want them to feel loved.	96.8%	2.5%	0.0%	0.6%
Your romantic partner doesn’t want to have sex with you, so you sexually touch a child instead.	96.8%	1.9%	1.3%	0.0%
You sexually touch a child to help them be less shy.	97.5%	1.3%	1.3%	0.0%

Next, the inter-item correlations for the remaining 14 items were examined to identify redundancy. Only two items were intercorrelated higher than .90 (“You let a child touch your penis for their sexual pleasure” and “A child wants to touch your penis, and you let them for your sexual pleasure”). We retained only the former item of this pair because it had the lower percentage of *very bad* responses. Intercorrelations for the retained 13 items are shown in Table 3. The internal consistency of the EASOC Scale was found to be excellent (α = .99). The final EASOC Scale is presented in the Appendix.

**Table 3:** Retained EASOC Scale Item Means, Standard Deviations, and Item Intercorrelations

Item	*M*	*SD*	1	2	3	4	5	6	7	8	9	10	11	12	13
1. You sexually touch a child.	1.09	0.35	–												
2. A child asks you about masturbation so you touch their genitals to teach them how to masturbate.	1.11	0.42	.69	–											
3. A child asks you about masturbation so you masturbate in front of the child to teach them how to do it.	1.11	0.43	.66	.86	–										
4. You are bathing a child and they ask you to wash their private parts. You wash and continue to rub their genitals to make the child feel good.	1.13	0.49	.75	.89	.89	–									
5. You let a child touch your penis for their sexual pleasure.	1.12	0.43	.66	.82	.76	.89	–								
6. You sexually touch a child to help them feel better about their body.	1.08	0.34	.76	.89	.86	.89	.72	–							
7. While you’re drunk, you sexually touch a child.	1.09	0.36	.65	.40	.39	.43	.46	.41	–						
8. You sexually touch a child because they won’t reject you like adults will.	1.09	0.33	.78	.82	.70	.76	.79	.74	.42	–					
9. Your partner wants to sexually touch a child with you, so you do it to make your partner happy.	1.10	0.36	.85	.73	.67	.71	.58	.72	.56	.74	–				
10. You are helping a child into their bathing suit, and you touch their genitals for your sexual pleasure.	1.11	0.39	.73	.83	.76	.85	.87	.70	.38	.82	.73	–			
11. A child puts their hands on your penis, and you let it continue for your sexual pleasure.	1.13	0.43	.69	.81	.78	.79	.78	.69	.37	.78	.69	.82	–		
12. A child sits on your lap and rubs against your penis, and you let it continue for your sexual pleasure.	1.13	0.45	.63	.85	.69	.82	.89	.65	.48	.75	.63	.86	.77	–	
13. You have a ‘tickle fight’ with a child and touch their genitals for your sexual pleasure.	1.11	0.44	.73	.63	.65	.76	.71	.67	.50	.64	.61	.67	.66	.64	–

### Association With Sexual Offending Against Children

We divided the participants into two groups: one group with sexual offenses against children (SOC; *n* = 58) and another group with no sexual offenses against children (No-SOC; *n* = 22). The SOC group consisted of those who had a current sexual offense against a child 12 years or younger, while the No-SOC group consisted of those who had a current non-sexual offense or who had a sexual offense against someone aged 16 years or older. Participants who did not report a current sexual offense against a child 12 years or younger while also reporting a prior sexual offense against a child 12 years or younger, a current or prior sexual offense against a child between 13 and 15 years of age, or a current or prior child pornography offense were not included in either group. This was done so that the SOC group had committed current contact sexual offenses against children and that the comparison No-SOC group had committed no child-related sexual offenses.

Due to the skewed nature of the data, Shapiro-Wilk’s test of normality was violated (*p* < .001) and the distributions of the SOC and No-SOC groups are not similar in shape. Therefore, a Mann-Whitney U comparing mean rank EASOC Scale total scores for the SOC and No-SOC groups was conducted. The results indicated that the SOC group (*M* = 1.20, *SD* = 0.44, scores ranged from 1.00 to 3.31) had significantly higher EASOC Scale scores than did the No-SOC group (*M* = 1.00, *SD* = .00, all scores were 1.00), *U* = 462.00, *p* < .001, *r* = .30.

## Discussion

The purpose of the current study was to take the first steps toward creating a self-report measure of evaluative attitudes toward sexual offending against children that would be sensitive and useful for determining the extent to which such evaluative attitudes may predict and explain sexual offending against children. We retained 13 of 30 items with the lowest endorsement of the most negative response option (i.e., *very bad*) and that were not too highly inter-correlated to create the Evaluative Attitudes Toward Sexual Offending Against Children (EASOC) Scale. As an initial test of the relevance of the EASOC Scale, we examined its association with sexual offending against children. Participants with sexual offenses against children reported more positive evaluative attitudes on the EASOC Scale than did those without sexual offenses against children. This expected association is a necessary (but not sufficient) indication that the EASOC Scale may be relevant for predicting and explaining sexual offending against children, and supports further research on the EASOC Scale with more rigorous methodologies.

There are a number of issues and limitations in our study that have implications for the interpretation of our findings and for future research. Endorsement of anything other than the most negative response option was very low even by participants who reported sexual offenses against children. Unexpectedly, the item with no potential mitigation (i.e., *You sexually touch a child*) was among the least skewed items and retained for inclusion in the EASOC Scale. This suggests that the contexts we included in the other items were not perceived as particularly mitigating by our participants. There was even less variance in the comparison group, with endorsement of only the most negative response option for all EASOC Scale items. Though this may be a sign that the EASOC Scale is working as it should, it was problematic for statistical analyses (e.g., may have exaggerated the magnitude of difference between groups) and may further indicate inadequate sensitivity of the EASOC Scale.

The observed extreme skewness may reflect a problem with the EASOC Scale, which would be consistent with feedback from several colleagues over the years warning us that almost nobody will report that they think there is anything positive about sexual offending against a child. Some of these same colleagues have argued that this is why cognitive distortion scales are useful for measuring evaluative attitudes—despite any conceptual ambiguity, they purportedly capture whether one believes that sexual offending against children is not that bad in a way that is more disarming and less socially undesirable than direct evaluations like the EASOC Scale items.

This may well be the case, but it may also or instead be something about our current sample of participants or data collection procedure. Participants’ responses were anonymous, and we made that clear instructionally and procedurally (e.g., no identifying information gathered, completed questionnaires submitted in sealed envelopes). Further, though data were collected by the Clinical Director of assessment and treatment statewide, she had minimal direct involvement with the participants prior to data collection (e.g., her office is not in the prison and she did not directly deliver clinical services to the participants). Nevertheless, it is still possible that participants did not trust that their responses would not be linked back to them and negatively impact them in some way.

The current study is a modest first attempt and future research with larger samples in different contexts (e.g., pre-treatment, prison, community, anonymous online surveys) should revisit item selection (e.g., item response theory or unique variable analysis; Christensen, Garrido, & Golino, 2023) and test the construct validity of EASOC Scale scores via factor analysis, experimental tests, convergence with and divergence from other measures of constructs presumed to be more or less similar, respectively, to evaluative attitudes toward sexual offending against children, and incremental correlations with sexual offending against children beyond cognitive distortion scales (Clark & Watson, 2019; Nunes et al., 2018, 2021, 2023; Pedneault et al., 2021). Future research may also want to refine the wording of some of the items to ensure they convey the intended meaning. Though we attempted to strike the best balance between clarity, conciseness, and simplicity, some of the items may be too complex, with multiple clauses, multiple sentences, or multiple concepts. Future research should also use more rigorous research designs (e.g., longitudinal, randomized experiments) to test the EASOC Scale’s scores relevance for predicting and explaining sexual offending against children (e.g., Harris & Rice, 2015; Heffernan et al., 2019; Nunes et al., 2019).

Though at least some aspects of cognitive distortion definitions and measures do not appear to us to conceptually fit neatly with evaluative attitudes, other constructs that have not usually been viewed under the attitude or cognitive distortion umbrella may be more related to or even overlapping with evaluative attitudes. One such construct is outcome expectancies. Outcome expectancies, in this context, refer to what one foresees as the consequences of committing a sexual offense and the extent to which those anticipated outcomes are viewed negatively versus positively. Outcome expectancies have been implicitly or explicitly hypothesized to play a role in sexual offending in a number of models, such as Relapse Prevention (Laws, 1989), Self-Regulation Model (Ward & Hudson, 1998), Judgement Model of Cognitive Distortions (Ward et al., 2006), and evolutionary explanations of rape (Lalumière et al., 2005). Though most of these models do not mention outcome expectancies by name, they invoke similar notions of anticipated benefits and costs influencing one’s likelihood of sexual offending.

If a person focuses on outcomes that they view as positive (e.g., sexual gratification) then we would expect they would have a positive evaluative attitude toward sexual offending. In contrast, if a person focuses on outcomes that they view as negative (e.g., harming children, shame, social ostracism, prison) then we would expect they would have a negative evaluative attitude toward sexual offending. This is consistent with a general social psychological model—the Expectancy-Value Model of Attitudes—in which perceived costs and benefits of a behavior inform one’s evaluative attitudes toward that behavior (Fishbein & Ajzen, 1975). Thus, it is possible that evaluative attitudes toward sexual offending against children have already been identified and addressed by researchers and practitioners, just using different names. It would be worthwhile to better integrate these ideas and measures of outcome expectancies, cognitive distortions, and evaluative attitudes to determine the extent to which they overlap and—if they are distinct—how they may work together to influence sexual offending.

## Supplementary Materials

The Supplementary Materials contain the following items (for access, see Nunes et al., 2023):

- Content of questionnaire administered to participants (demographic questions and main questionnaire)
- Response frequencies for initial EASOC Scale items with the negative response scale (Table S1)

NunesK. L.
HawthornD. M. L.
BatemanE. R.
GriffithA. L.
FraserJ. M.
 (2023). Supplementary materials to "First steps in the development of a new measure of attitudes toward sexual offending against children"
[Questionnaire and additional information]. PsychOpen. 10.23668/psycharchives.13968
PMC1177874139885890

## Data Availability

The dataset is available upon request.

## Appendix: Evaluative Attitudes Toward Sexual Offending Against Children (EASOC) Scale

Please rate how you think or feel about each of the items below. For example, how good or bad do you think it would be to do something sexual with a child?

For all the items below, “child” means someone 13 years old or younger.

Answering these questions does NOT mean that you have done or will do the things described in the questions. Please just say what you think about the idea of doing each of these things, even if you have never done them or never will do them.

**Table:** Please circle one of the four answers under each statement.

RESPONSE SCALE							
	very bad		pretty bad		not so bad		not bad at all

1. You sexually touch a child.
2. A child asks you about masturbation so you touch their genitals to teach them how to masturbate.
3. A child asks about masturbation so you masturbate in front of the child to teach them how to do it.
4. You are bathing a child and they ask you to wash their private parts. You wash and continue to rub their genitals to make the child feel good.
5. You let a child touch your penis for their sexual pleasure.
6. You sexually touch a child to help them feel better about their body.
7. While you’re drunk, you sexually touch a child.
8. You sexually touch a child because they won’t reject you like adults will.
9. Your partner wants to sexually touch a child with you, so you do it to make your partner happy.
10. You are helping a child into their bathing suit, and you touch their genitals for your sexual pleasure.
11. A child puts their hand on your penis, and you let it continue for your sexual pleasure.
12. A child sits on your lap and rubs against your penis, you let it continue for your sexual pleasure.
13. You have a “tickle fight” with a child and touch their genitals for your sexual pleasure.

## Related Versions

The current data were previously used for Emily Bateman’s master’s thesis (Dunn, 2021).
